# Acetonitrile­triaqua­[3-eth­oxy-1,8-(3,6,9-trioxaundecane-1,11-diyldi­oxy)-9*H*-xanthen-9-one]terbium(III) tris­(perchlorate)

**DOI:** 10.1107/S1600536810018313

**Published:** 2010-05-22

**Authors:** Wen-Jie He, Xiao-Bo Pan, Li-Hui Yao, Bing-Ran Yu, Jin-Cai Wu, Ning Tang

**Affiliations:** a College of Chemistry and Chemical Engineering, Lanzhou University, Lanzhou 730000, People’s Republic of China

## Abstract

In the title compound, [Tb(CH_3_CN)(C_23_H_26_O_8_)(H_2_O)_3_](ClO_4_)_3_, the Tb^3+^ atom is eight-coordinated by one N atom of an acetonitrile molecule, three water O atoms and four ligand O atoms. The Tb^3+^ atom is located on one side of the macrocycle and the carbonyl oxygen coordinated to the terbium [Tb1—O1= 2.210 (3) Å] is bent out of the xanthone plane by 0.514 (3) Å. The geometry around terbium is a distorted two-capped trigonal prism.

## Related literature

For a previous study of xanthone–ether, see: Shen, Pan, Wang, Wu *et al.* (2008 ▶); Wu *et al.* (2009 ▶). For the synthesis of similar xanthone–ether compounds, see: Shen, Pan, Wang, Yao *et al.* (2008 ▶); Mills *et al.* (1995 ▶).
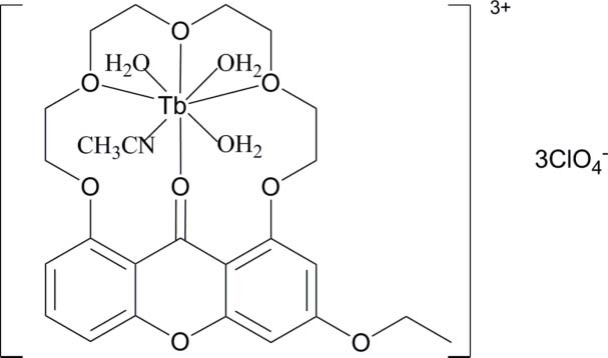


## Experimental

### 

#### Crystal data

[Tb(C_2_H_3_N)(C_23_H_26_O_8_)(H_2_O)_3_](ClO_4_)_3_
*M* *_r_* = 982.81
Triclinic, 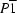

*a* = 10.2838 (2) Å
*b* = 11.7932 (3) Å
*c* = 15.4680 (4) Å
α = 85.933 (1)°
β = 84.813 (1)°
γ = 77.363 (1)°
*V* = 1820.48 (7) Å^3^
*Z* = 2
Mo *K*α radiation
μ = 2.25 mm^−1^
*T* = 296 K
0.25 × 0.21 × 0.15 mm

#### Data collection

Bruker SMART CCD area-detector diffractometer
Absorption correction: multi-scan (*SADABS*; Bruker, 2002 ▶) *T*_min_ = 0.575, *T*_max_ = 0.713
11523 measured reflections
8058 independent reflections
6378 reflections with *I* > 2σ(*I*)
*R*_int_ = 0.019

#### Refinement

*R*[*F* ^2^ > 2σ(*F* ^2^)] = 0.044
*wR*(*F* ^2^) = 0.113
*S* = 1.03
8058 reflections
495 parameters
H atoms treated by a mixture of independent and constrained refinement
Δρ_max_ = 0.96 e Å^−3^
Δρ_min_ = −0.85 e Å^−3^

### 

Data collection: *SMART* (Bruker, 2002 ▶); cell refinement: *SAINT* (Bruker, 2002 ▶); data reduction: *SAINT*; program(s) used to solve structure: *SHELXS97* (Sheldrick, 2008 ▶); program(s) used to refine structure: *SHELXL97* (Sheldrick, 2008 ▶); molecular graphics: *SHELXTL* (Sheldrick, 2008 ▶); software used to prepare material for publication: *SHELXTL*.

## Supplementary Material

Crystal structure: contains datablocks I, global. DOI: 10.1107/S1600536810018313/kp2258sup1.cif

Structure factors: contains datablocks I. DOI: 10.1107/S1600536810018313/kp2258Isup2.hkl

Additional supplementary materials:  crystallographic information; 3D view; checkCIF report

## Figures and Tables

**Table 1:** Selected bond lengths (Å)

Tb1—O1	2.210 (3)
Tb1—O23	2.359 (5)
Tb1—O22	2.391 (4)
Tb1—O21	2.412 (4)
Tb1—O4	2.442 (4)
Tb1—N1	2.467 (5)
Tb1—O3	2.467 (4)
Tb1—O5	2.477 (4)

**Fig. 1.**
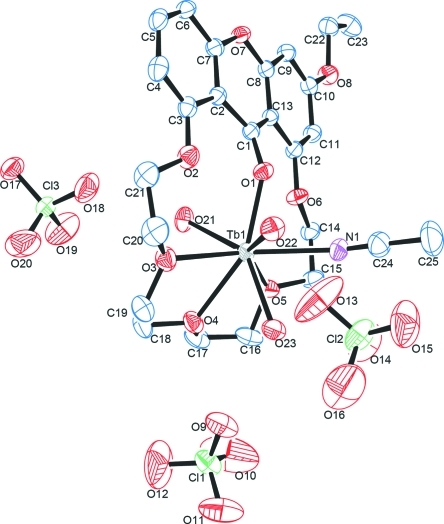
Thermal ellipsoid (30%) diagram of the title compound. Hydrogen atoms have been excluded for clarity.

**Fig. 2.**
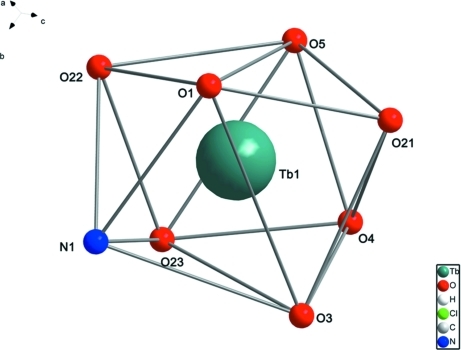
The eight-coordination with a distorted two-capped trigonal prism around terbium in the title compound.

## supplementary crystallographic information

### Comment

The xanthone derivatives show good properties in pharmacology and selectively recognition of guest species. A series of alkali metal and alkaline earth metal complexes derived from xanthone-crown ether have been synthesised and studied as fluorescent sensors. However, the rare earth complexes with novel structure derived from xanthone-crown ether have rarely been reported. Herein, we report the synthesis and structure of the title compound, 3-ethoxy-1,8-(3,6,9-trioxaundecane-1,11-diylioxy)xanthone terbium(III) perchlorate. The Tb^3+^ is located on one side of the macrocycle and the carbonyl oxygen coordinated to the terbium (Tb1—O1= 2.210 (3) Å) is bent out of the xanthone plane as to increase the coordination space. Tb^3+^ is eight coordinated by one nitrogen of CH_3_CN (Tb1—N1= 2.467 (5) Å), three oxygens from three water molecule (Tb1—O21= 2.412 (4) Å, Tb1—O22= 2.391 (4) Å, Tb1—O23= 2.359 (5) Å) and four ligand oxygens (Tb1—O1= 2.210 (3) Å, Tb1—O3= 2.467 (4) Å, Tb1—O4= 2.442 (4) Å, and Tb1—O5= 2.477 (4) Å) (Fig. 1 and Table 1). The selected bond angles around Tb^3+^ were listed as following: O1—Tb1—O3 = 105.85°, O1—Tb1—O4 = 148.44°, O1—Tb1—O5 = 106.73°, O3—Tb1—O4 = 66.29°, O4—Tb1—O5 = 66.27°, O3—Tb1—O5 = 130.40°, O21—Tb1—O22 = 132.39°, O21—Tb1—O23 = 141.50°, O22—Tb1—O23 = 75.57°. Geometry around terbium is a distorted two-capped trigonal prism.

### Experimental

3-ethoxy-1,8-trihydroxyxanthone was prepared as follows: 1,3,8-trihydroxyxanthone (2.44 g, 10 mmol) was dissolved in acethone (150 ml). Bromoethane (1.64 g, 15 mmol) and anhydrous potassium carbonate (2 g) was added. Then the mixture was stirred at 333 K for 12 h. The resulting mixture was filtrated and the filtration was evaporated.The residue was purified by column chromatography(SiO_2_, EtOAc/petroleum ether, 1:9). Then light-yellow powder was obtained. Yield: 61.20%. MS (ESI) m/z(%): 272.0 [M], ^1^H NMR(300 MHz, CDCl_3_): 7.60-7.51 (t, 1 H), 6.89-6.76 (m, 2 H), 6.40-6.17 (d, 2 H), 4.18-4.07(m, 2 H), 1.56-1.43(m, 3 H).

3-Ethoxy-1,8-(3,6,9-trioxaundecane-1,11-diylioxy)xanthone was prepared as follows: 3-ethoxy-1,8-trihydroxyxanthone (1.36 g, 5 mmol) was dissolved in the dry DMF (350 ml), and anhydrous potassium carbonate (2.07 g,15 mmol) was added under N_2_. 1,11-Dibromo-3,6,9-trioxaundecane (3.20 g, 10 mmol) was added and the mixture refluxed for 14 h. Most of the DMF was evaporated. The resulting mixture was diluted with water (60 ml), extracted several times with chloroform and the chloroform extracts evaporated. The residue was purified by column chromatography (SiO_2_, CHCl_3_/EtOH, 10:1), and then recrystallized from dry toluene afforded L as light-yellow crystals. Yield: 40.60%. M.p. 434-436 K. MS (ESI) m/z(%): 430.3 [M]. ^1^H NMR (400 MHz, CDCl_3_, ppm): 7.45-7.41 (t, 1H); 6.91-6.89 (d, 1H); 6.68-6.66 (d, 1H); 6.38-6.37 (s, 1H); 6.24-6.23 (s, 1H); 4.20-4.14 (m, 4H); 4.02-3.97 (m, 8 H); 3.85-3.81 (m, 4 H); 4.20-4.14 (m, 4H); 4.11-4.05 (m, 2H); 1.46-1.42 (m, 3H); IR (KBr, cm^-1^): 3426(s), 2869(s), 1661(s), 1566(s), 1473(s), 1322(s), 1268(s), 1114(s), 893(s), 772(s).

The title compound was prepared as follows: ligand (86.1 mg, 0.2 mmol) was dissolved in 7 ml of acetonitrile. Terbium(III) perchlorate hexahydrate (113.1 mg, 0.2 mmol) was dissolved in 4 ml of acetonitrile and added dropwise to the above solution. After the solution was stirred for 2 h, all the solvent was evaporated. The residue was redissolved in 3 ml of acetonitrile and layered with diethyl ether. Fine yellowish-block crystal was obtained. Yield: 50%. M.p. 447-449 K. Elemental anal. calcd for C_25_H_35_O_23_Cl_3_NTb: C, 30.55%; H, 3.59%; N, 1.43%. Found: C, 30.70%; H, 3.41%; N, 1.59%. IR (KBr, cm^-1^): 3357(s), 2934(s), 2869(s), 1625(s), 1564(s), 1474(s), 1319(s), 1273(s), 1112(s), 889(s), 778(s).

### Crystal data

**Table:**

[Tb(C_2_H_3_N)(C_23_H_26_O_8_)(H_2_O)_3_](ClO_4_)_3_	*Z* = 2
*M**_r_* = 982.81	*F*(000) = 984
Triclinic, *P*1	*D*_x_ = 1.793 Mg m^−^^3^
Hall symbol: -P 1	Mo *K*α radiation, λ = 0.71073 Å
*a* = 10.2838 (2) Å	Cell parameters from 5453 reflections
*b* = 11.7932 (3) Å	θ = 1.3–27.6°
*c* = 15.4680 (4) Å	µ = 2.25 mm^−^^1^
α = 85.933 (1)°	*T* = 296 K
β = 84.813 (1)°	Block, light yellow
γ = 77.363 (1)°	0.25 × 0.21 × 0.15 mm
*V* = 1820.48 (7) Å^3^	

### Data collection

**Table:**

Bruker SMART CCD area-detector diffractometer	8058 independent reflections
Radiation source: fine-focus sealed tube	6378 reflections with *I* > 2σ(*I*)
graphite	*R*_int_ = 0.019
phi and ω scans	θ_max_ = 27.6°, θ_min_ = 1.3°
Absorption correction: multi-scan (*SADABS*; Bruker, 2002)	*h* = −13→11
*T*_min_ = 0.575, *T*_max_ = 0.713	*k* = −13→15
11523 measured reflections	*l* = −20→20

### Refinement

**Table:**

Refinement on *F*^2^	Primary atom site location: structure-invariant direct methods
Least-squares matrix: full	Secondary atom site location: difference Fourier map
*R*[*F*^2^ > 2σ(*F*^2^)] = 0.044	Hydrogen site location: inferred from neighbouring sites
*wR*(*F*^2^) = 0.113	H atoms treated by a mixture of independent and constrained refinement
*S* = 1.03	*w* = 1/[σ^2^(*F*_o_^2^) + (0.0551*P*)^2^ + 2.2597*P*] where *P* = (*F*_o_^2^ + 2*F*_c_^2^)/3
8058 reflections	(Δ/σ)_max_ = 0.001
495 parameters	Δρ_max_ = 0.96 e Å^−^^3^
0 restraints	Δρ_min_ = −0.85 e Å^−^^3^

### Special details

**Table:**

Geometry. All esds (except the esd in the dihedral angle between two l.s. planes) are estimated using the full covariance matrix. The cell esds are taken into account individually in the estimation of esds in distances, angles and torsion angles; correlations between esds in cell parameters are only used when they are defined by crystal symmetry. An approximate (isotropic) treatment of cell esds is used for estimating esds involving l.s. planes.
Refinement. Refinement of *F*^2^ against ALL reflections. The weighted *R*-factor wR and goodness of fit *S* are based on *F*^2^, conventional *R*-factors *R* are based on *F*, with *F* set to zero for negative *F*^2^. The threshold expression of *F*^2^ > σ(*F*^2^) is used only for calculating *R*-factors(gt) etc. and is not relevant to the choice of reflections for refinement. *R*-factors based on *F*^2^ are statistically about twice as large as those based on *F*, and *R*- factors based on ALL data will be even larger.

### Fractional atomic coordinates and isotropic or equivalent isotropic displacement parameters (Å^2^)

**Table:**

	*x*	*y*	*z*	*U*_iso_*/*U*_eq_	
Tb1	0.62956 (3)	0.68996 (2)	0.713567 (15)	0.04133 (9)	
O1	0.8255 (3)	0.6826 (3)	0.7643 (2)	0.0450 (8)	
O21	0.6216 (5)	0.5850 (4)	0.8529 (3)	0.0566 (10)	
H21	0.6430	0.5149	0.8460	0.085*	
O3	0.4659 (3)	0.8280 (3)	0.8027 (2)	0.0532 (9)	
O4	0.4088 (4)	0.6427 (4)	0.7352 (2)	0.0583 (10)	
O22	0.7823 (5)	0.6600 (4)	0.5871 (3)	0.0629 (11)	
H22	0.7403	0.6693	0.5436	0.094*	
O5	0.6272 (5)	0.4898 (4)	0.6758 (3)	0.0645 (11)	
Cl3	0.58766 (14)	0.28177 (12)	0.93099 (9)	0.0524 (3)	
O7	1.1022 (3)	0.5922 (3)	0.9413 (2)	0.0450 (8)	
O2	0.6953 (3)	0.8444 (3)	0.8646 (2)	0.0479 (8)	
O23	0.4973 (5)	0.7627 (6)	0.5967 (3)	0.0722 (16)	
H23	0.5268	0.8151	0.5696	0.108*	
C8	1.0050 (5)	0.6910 (4)	0.9512 (3)	0.0419 (11)	
C1	0.8996 (5)	0.6481 (4)	0.8254 (3)	0.0394 (11)	
C12	0.7988 (5)	0.8197 (4)	0.9165 (3)	0.0426 (11)	
C14	0.5776 (5)	0.9279 (5)	0.8924 (4)	0.0479 (12)	
H14A	0.6002	1.0018	0.9008	0.058*	
H14B	0.5375	0.9006	0.9469	0.058*	
C15	0.4839 (6)	0.9414 (5)	0.8238 (4)	0.0567 (14)	
H15A	0.3984	0.9890	0.8436	0.068*	
H15B	0.5186	0.9803	0.7723	0.068*	
Cl1	0.12202 (18)	0.72796 (18)	0.58648 (11)	0.0746 (5)	
O6	0.8842 (4)	0.4699 (3)	0.7159 (3)	0.0592 (10)	
O17	0.6598 (4)	0.1902 (4)	0.9828 (3)	0.0781 (14)	
O9	0.2559 (5)	0.7073 (6)	0.5564 (3)	0.0971 (17)	
C2	0.9915 (5)	0.5367 (4)	0.8252 (3)	0.0400 (11)	
C3	0.9880 (5)	0.4490 (5)	0.7672 (3)	0.0474 (12)	
C11	0.8093 (5)	0.8861 (5)	0.9839 (3)	0.0456 (12)	
H11	0.7421	0.9505	0.9973	0.055*	
C7	1.0918 (5)	0.5144 (5)	0.8830 (3)	0.0435 (11)	
O19	0.4947 (7)	0.3609 (6)	0.9798 (4)	0.119 (2)	
C6	1.1903 (5)	0.4127 (5)	0.8834 (4)	0.0508 (13)	
H6	1.2562	0.3994	0.9226	0.061*	
C22	1.0381 (6)	0.9085 (5)	1.1451 (4)	0.0578 (14)	
H22A	1.1163	0.9158	1.1069	0.069*	
H22B	1.0533	0.8307	1.1725	0.069*	
O8	0.9219 (4)	0.9290 (3)	1.0964 (2)	0.0556 (9)	
N1	0.6831 (5)	0.8781 (4)	0.6609 (3)	0.0579 (12)	
O18	0.6795 (6)	0.3394 (5)	0.8823 (4)	0.111 (2)	
C16	0.3279 (6)	0.8247 (7)	0.7959 (4)	0.0669 (18)	
H16A	0.2983	0.8624	0.7412	0.080*	
H16B	0.2723	0.8656	0.8429	0.080*	
C4	1.0872 (6)	0.3490 (5)	0.7670 (4)	0.0590 (15)	
H4	1.0872	0.2921	0.7283	0.071*	
C5	1.1868 (6)	0.3329 (5)	0.8241 (4)	0.0594 (16)	
H5	1.2536	0.2655	0.8220	0.071*	
C24	0.7305 (8)	0.9440 (7)	0.6198 (5)	0.0745 (19)	
C17	0.3163 (6)	0.7015 (7)	0.8009 (4)	0.0716 (19)	
H17A	0.3369	0.6659	0.8579	0.086*	
H17B	0.2259	0.6964	0.7912	0.086*	
C23	1.0136 (7)	0.9969 (7)	1.2126 (4)	0.077 (2)	
H23A	0.9924	1.0735	1.1851	0.115*	
H23B	1.0923	0.9889	1.2434	0.115*	
H23C	0.9402	0.9851	1.2527	0.115*	
C18	0.4008 (8)	0.5229 (6)	0.7296 (5)	0.085 (2)	
H18A	0.3113	0.5184	0.7173	0.102*	
H18B	0.4213	0.4798	0.7842	0.102*	
C20	0.7348 (8)	0.4169 (6)	0.6267 (5)	0.082 (2)	
H20A	0.7610	0.4606	0.5751	0.098*	
H20B	0.7039	0.3511	0.6082	0.098*	
C19	0.4985 (8)	0.4731 (7)	0.6589 (5)	0.083 (2)	
H19A	0.5007	0.3907	0.6564	0.099*	
H19B	0.4730	0.5114	0.6035	0.099*	
C21	0.8518 (8)	0.3737 (6)	0.6776 (5)	0.0717 (18)	
H21A	0.8319	0.3173	0.7227	0.086*	
H21B	0.9272	0.3360	0.6401	0.086*	
O20	0.5193 (8)	0.2364 (6)	0.8729 (6)	0.155 (3)	
O11	0.0459 (7)	0.7624 (8)	0.5156 (5)	0.150 (3)	
Cl2	0.6453 (2)	0.83662 (18)	0.38551 (11)	0.0855 (6)	
O14	0.5774 (8)	0.8979 (6)	0.4544 (4)	0.124 (2)	
O13	0.7159 (10)	0.7302 (8)	0.4167 (4)	0.191 (5)	
O12	0.0867 (10)	0.6318 (8)	0.6195 (8)	0.214 (5)	
C25	0.7899 (11)	1.0288 (9)	0.5657 (7)	0.123 (4)	
H25A	0.8034	1.0060	0.5066	0.185*	
H25B	0.8742	1.0320	0.5864	0.185*	
H25C	0.7311	1.1040	0.5684	0.185*	
O15	0.7179 (9)	0.8940 (7)	0.3332 (6)	0.169 (4)	
O16	0.5489 (14)	0.8129 (8)	0.3300 (7)	0.228 (6)	
O10	0.0952 (10)	0.8067 (11)	0.6477 (7)	0.236 (6)	
C13	0.8999 (5)	0.7188 (4)	0.8973 (3)	0.0383 (10)	
C10	0.9210 (5)	0.8566 (5)	1.0321 (3)	0.0450 (12)	
C9	1.0207 (5)	0.7590 (5)	1.0162 (3)	0.0458 (12)	
H9	1.0954	0.7402	1.0483	0.055*	
H21C	0.569 (6)	0.614 (5)	0.888 (4)	0.049 (18)*	
H22C	0.868 (7)	0.639 (6)	0.588 (5)	0.08 (2)*	
H23D	0.447 (8)	0.747 (8)	0.595 (6)	0.09 (4)*	

### Atomic displacement parameters (Å^2^)

**Table:**

	*U*^11^	*U*^22^	*U*^33^	*U*^12^	*U*^13^	*U*^23^
Tb1	0.04631 (15)	0.04543 (15)	0.03356 (13)	−0.01557 (11)	−0.00238 (9)	0.00619 (9)
O1	0.0435 (19)	0.050 (2)	0.0392 (18)	−0.0054 (16)	−0.0057 (15)	0.0032 (15)
O21	0.079 (3)	0.049 (2)	0.040 (2)	−0.017 (2)	0.007 (2)	0.0028 (17)
O3	0.0391 (19)	0.064 (3)	0.057 (2)	−0.0079 (18)	−0.0062 (16)	−0.0111 (19)
O4	0.060 (2)	0.070 (3)	0.052 (2)	−0.033 (2)	−0.0022 (18)	0.0069 (19)
O22	0.064 (3)	0.074 (3)	0.047 (2)	−0.012 (2)	0.003 (2)	0.004 (2)
O5	0.077 (3)	0.053 (3)	0.068 (3)	−0.022 (2)	−0.011 (2)	−0.006 (2)
Cl3	0.0543 (8)	0.0476 (8)	0.0520 (7)	−0.0045 (6)	−0.0053 (6)	0.0023 (6)
O7	0.0366 (18)	0.045 (2)	0.049 (2)	−0.0004 (15)	−0.0050 (15)	0.0018 (16)
O2	0.0410 (19)	0.048 (2)	0.049 (2)	0.0060 (16)	−0.0106 (15)	−0.0079 (16)
O23	0.062 (3)	0.107 (4)	0.055 (3)	−0.042 (3)	−0.018 (2)	0.032 (2)
C8	0.038 (3)	0.042 (3)	0.045 (3)	−0.012 (2)	0.001 (2)	0.005 (2)
C1	0.034 (2)	0.042 (3)	0.039 (2)	−0.008 (2)	0.0051 (19)	0.003 (2)
C12	0.042 (3)	0.039 (3)	0.045 (3)	−0.006 (2)	−0.004 (2)	0.003 (2)
C14	0.045 (3)	0.038 (3)	0.057 (3)	0.001 (2)	−0.004 (2)	−0.009 (2)
C15	0.049 (3)	0.051 (3)	0.064 (4)	0.007 (3)	−0.014 (3)	−0.007 (3)
Cl1	0.0713 (10)	0.0992 (13)	0.0619 (9)	−0.0366 (10)	0.0023 (8)	−0.0143 (9)
O6	0.070 (3)	0.047 (2)	0.060 (2)	−0.008 (2)	−0.009 (2)	−0.0096 (18)
O17	0.063 (3)	0.074 (3)	0.089 (3)	−0.006 (2)	−0.005 (2)	0.030 (3)
O9	0.074 (3)	0.150 (5)	0.080 (3)	−0.048 (3)	−0.009 (3)	−0.012 (3)
C2	0.039 (3)	0.040 (3)	0.039 (2)	−0.008 (2)	0.0040 (19)	0.002 (2)
C3	0.050 (3)	0.045 (3)	0.045 (3)	−0.010 (2)	0.004 (2)	0.000 (2)
C11	0.044 (3)	0.042 (3)	0.048 (3)	−0.004 (2)	−0.001 (2)	−0.003 (2)
C7	0.038 (3)	0.049 (3)	0.042 (3)	−0.011 (2)	0.003 (2)	0.005 (2)
O19	0.126 (5)	0.115 (5)	0.086 (4)	0.032 (4)	0.021 (4)	−0.015 (3)
C6	0.038 (3)	0.052 (3)	0.058 (3)	−0.003 (2)	−0.001 (2)	0.010 (3)
C22	0.061 (4)	0.061 (4)	0.056 (3)	−0.019 (3)	−0.013 (3)	−0.004 (3)
O8	0.061 (2)	0.053 (2)	0.054 (2)	−0.0103 (19)	−0.0105 (18)	−0.0107 (18)
N1	0.067 (3)	0.052 (3)	0.055 (3)	−0.017 (2)	−0.010 (2)	0.012 (2)
O18	0.088 (4)	0.088 (4)	0.139 (5)	−0.005 (3)	0.017 (4)	0.051 (4)
C16	0.038 (3)	0.109 (6)	0.056 (4)	−0.019 (3)	0.000 (2)	−0.015 (4)
C4	0.066 (4)	0.045 (3)	0.060 (4)	−0.005 (3)	0.015 (3)	−0.005 (3)
C5	0.048 (3)	0.043 (3)	0.077 (4)	0.000 (3)	0.012 (3)	0.008 (3)
C24	0.085 (5)	0.077 (5)	0.064 (4)	−0.026 (4)	−0.011 (4)	0.008 (4)
C17	0.051 (3)	0.114 (6)	0.056 (4)	−0.038 (4)	0.006 (3)	0.006 (4)
C23	0.074 (4)	0.099 (6)	0.067 (4)	−0.034 (4)	−0.007 (3)	−0.023 (4)
C18	0.085 (5)	0.081 (5)	0.102 (6)	−0.053 (4)	−0.014 (4)	0.023 (4)
C20	0.107 (6)	0.062 (4)	0.077 (5)	−0.009 (4)	−0.011 (4)	−0.037 (4)
C19	0.090 (5)	0.067 (5)	0.103 (6)	−0.036 (4)	−0.022 (5)	−0.008 (4)
C21	0.091 (5)	0.058 (4)	0.065 (4)	−0.007 (4)	−0.009 (4)	−0.020 (3)
O20	0.171 (7)	0.114 (5)	0.189 (8)	−0.001 (5)	−0.114 (6)	−0.037 (5)
O11	0.102 (5)	0.235 (9)	0.121 (6)	−0.028 (5)	−0.047 (4)	−0.036 (6)
Cl2	0.1145 (15)	0.0836 (13)	0.0432 (8)	0.0040 (11)	0.0072 (9)	0.0045 (8)
O14	0.175 (7)	0.107 (5)	0.070 (4)	0.005 (4)	0.022 (4)	−0.016 (3)
O13	0.240 (10)	0.175 (8)	0.080 (4)	0.104 (7)	0.019 (5)	0.026 (5)
O12	0.176 (8)	0.158 (8)	0.289 (12)	−0.062 (7)	0.077 (8)	0.081 (8)
C25	0.149 (9)	0.114 (8)	0.122 (8)	−0.073 (7)	−0.010 (7)	0.033 (6)
O15	0.168 (8)	0.130 (6)	0.188 (8)	−0.038 (6)	0.070 (6)	0.036 (6)
O16	0.378 (17)	0.152 (8)	0.186 (9)	−0.063 (9)	−0.161 (11)	−0.016 (7)
O10	0.158 (8)	0.340 (15)	0.215 (10)	0.000 (9)	−0.021 (7)	−0.211 (11)
C13	0.037 (2)	0.038 (3)	0.039 (2)	−0.007 (2)	−0.0002 (19)	0.0036 (19)
C10	0.048 (3)	0.046 (3)	0.044 (3)	−0.016 (2)	−0.002 (2)	−0.001 (2)
C9	0.043 (3)	0.050 (3)	0.045 (3)	−0.011 (2)	−0.007 (2)	0.002 (2)

### Geometric parameters (Å, °)

**Table:**

Tb1—O1	2.210 (3)	C2—C7	1.395 (7)
Tb1—O23	2.359 (5)	C2—C3	1.424 (7)
Tb1—O22	2.391 (4)	C3—C4	1.381 (8)
Tb1—O21	2.412 (4)	C11—C10	1.393 (7)
Tb1—O4	2.442 (4)	C11—H11	0.9300
Tb1—N1	2.467 (5)	C7—C6	1.391 (7)
Tb1—O3	2.467 (4)	C6—C5	1.368 (8)
Tb1—O5	2.477 (4)	C6—H6	0.9300
O1—C1	1.257 (6)	C22—O8	1.437 (7)
O21—H21	0.8200	C22—C23	1.494 (8)
O21—H21C	0.78 (6)	C22—H22A	0.9700
O3—C16	1.442 (6)	C22—H22B	0.9700
O3—C15	1.453 (7)	O8—C10	1.358 (6)
O4—C17	1.433 (7)	N1—C24	1.134 (8)
O4—C18	1.443 (8)	C16—C17	1.480 (10)
O22—H22	0.8200	C16—H16A	0.9700
O22—H22C	0.86 (7)	C16—H16B	0.9700
O5—C19	1.430 (8)	C4—C5	1.386 (9)
O5—C20	1.437 (8)	C4—H4	0.9300
Cl3—O19	1.391 (5)	C5—H5	0.9300
Cl3—O20	1.391 (6)	C24—C25	1.459 (10)
Cl3—O17	1.411 (5)	C17—H17A	0.9700
Cl3—O18	1.415 (5)	C17—H17B	0.9700
O7—C7	1.357 (6)	C23—H23A	0.9600
O7—C8	1.366 (6)	C23—H23B	0.9600
O2—C12	1.359 (6)	C23—H23C	0.9600
O2—C14	1.437 (6)	C18—C19	1.478 (11)
O23—H23	0.8200	C18—H18A	0.9700
O23—H23D	0.59 (8)	C18—H18B	0.9700
C8—C9	1.372 (7)	C20—C21	1.474 (10)
C8—C13	1.393 (7)	C20—H20A	0.9700
C1—C13	1.436 (7)	C20—H20B	0.9700
C1—C2	1.441 (7)	C19—H19A	0.9700
C12—C11	1.373 (7)	C19—H19B	0.9700
C12—C13	1.428 (7)	C21—H21A	0.9700
C14—C15	1.474 (7)	C21—H21B	0.9700
C14—H14A	0.9700	Cl2—O15	1.306 (7)
C14—H14B	0.9700	Cl2—O14	1.378 (5)
C15—H15A	0.9700	Cl2—O13	1.383 (7)
C15—H15B	0.9700	Cl2—O16	1.452 (9)
Cl1—O12	1.321 (8)	C25—H25A	0.9600
Cl1—O10	1.341 (7)	C25—H25B	0.9600
Cl1—O9	1.387 (5)	C25—H25C	0.9600
Cl1—O11	1.390 (7)	C10—C9	1.383 (7)
O6—C3	1.358 (7)	C9—H9	0.9300
O6—C21	1.428 (7)		
			
O1—Tb1—O23	143.59 (15)	C4—C3—C2	119.1 (5)
O1—Tb1—O22	76.61 (15)	C12—C11—C10	119.8 (5)
O23—Tb1—O22	75.57 (18)	C12—C11—H11	120.1
O1—Tb1—O21	74.85 (14)	C10—C11—H11	120.1
O23—Tb1—O21	141.50 (17)	O7—C7—C6	115.2 (5)
O22—Tb1—O21	132.39 (16)	O7—C7—C2	122.2 (5)
O1—Tb1—O4	148.44 (13)	C6—C7—C2	122.5 (5)
O23—Tb1—O4	67.93 (16)	C5—C6—C7	117.6 (5)
O22—Tb1—O4	127.08 (15)	C5—C6—H6	121.2
O21—Tb1—O4	73.59 (15)	C7—C6—H6	121.2
O1—Tb1—N1	76.60 (15)	O8—C22—C23	107.7 (5)
O23—Tb1—N1	72.53 (18)	O8—C22—H22A	110.2
O22—Tb1—N1	71.28 (17)	C23—C22—H22A	110.2
O21—Tb1—N1	135.07 (15)	O8—C22—H22B	110.2
O4—Tb1—N1	127.39 (16)	C23—C22—H22B	110.2
O1—Tb1—O3	105.85 (12)	H22A—C22—H22B	108.5
O23—Tb1—O3	86.31 (18)	C10—O8—C22	117.9 (4)
O22—Tb1—O3	147.85 (15)	C24—N1—Tb1	160.0 (6)
O21—Tb1—O3	77.27 (15)	O3—C16—C17	108.4 (5)
O4—Tb1—O3	66.29 (13)	O3—C16—H16A	110.0
N1—Tb1—O3	78.08 (15)	C17—C16—H16A	110.0
O1—Tb1—O5	106.73 (14)	O3—C16—H16B	110.0
O23—Tb1—O5	89.00 (19)	C17—C16—H16B	110.0
O22—Tb1—O5	76.39 (15)	H16A—C16—H16B	108.4
O21—Tb1—O5	76.40 (14)	C3—C4—C5	120.3 (6)
O4—Tb1—O5	66.27 (15)	C3—C4—H4	119.8
N1—Tb1—O5	145.78 (16)	C5—C4—H4	119.8
O3—Tb1—O5	130.40 (14)	C6—C5—C4	122.3 (6)
C1—O1—Tb1	146.5 (3)	C6—C5—H5	118.8
Tb1—O21—H21	109.5	C4—C5—H5	118.8
Tb1—O21—H21C	117 (5)	N1—C24—C25	178.9 (9)
H21—O21—H21C	124.8	O4—C17—C16	107.8 (5)
C16—O3—C15	113.1 (4)	O4—C17—H17A	110.1
C16—O3—Tb1	115.2 (3)	C16—C17—H17A	110.1
C15—O3—Tb1	124.5 (3)	O4—C17—H17B	110.1
C17—O4—C18	113.2 (5)	C16—C17—H17B	110.1
C17—O4—Tb1	117.7 (3)	H17A—C17—H17B	108.5
C18—O4—Tb1	117.8 (4)	C22—C23—H23A	109.5
Tb1—O22—H22	109.5	C22—C23—H23B	109.5
Tb1—O22—H22C	125 (5)	H23A—C23—H23B	109.5
H22—O22—H22C	125.5	C22—C23—H23C	109.5
C19—O5—C20	113.0 (5)	H23A—C23—H23C	109.5
C19—O5—Tb1	114.3 (4)	H23B—C23—H23C	109.5
C20—O5—Tb1	123.9 (4)	O4—C18—C19	107.9 (5)
O19—Cl3—O20	107.9 (5)	O4—C18—H18A	110.1
O19—Cl3—O17	112.7 (4)	C19—C18—H18A	110.1
O20—Cl3—O17	109.5 (4)	O4—C18—H18B	110.1
O19—Cl3—O18	109.9 (4)	C19—C18—H18B	110.1
O20—Cl3—O18	108.0 (5)	H18A—C18—H18B	108.4
O17—Cl3—O18	108.5 (3)	O5—C20—C21	112.1 (5)
C7—O7—C8	120.1 (4)	O5—C20—H20A	109.2
C12—O2—C14	118.2 (4)	C21—C20—H20A	109.2
Tb1—O23—H23	109.5	O5—C20—H20B	109.2
Tb1—O23—H23D	118 (9)	C21—C20—H20B	109.2
H23—O23—H23D	131.4	H20A—C20—H20B	107.9
O7—C8—C9	115.1 (5)	O5—C19—C18	108.4 (6)
O7—C8—C13	121.1 (4)	O5—C19—H19A	110.0
C9—C8—C13	123.8 (5)	C18—C19—H19A	110.0
O1—C1—C13	121.9 (4)	O5—C19—H19B	110.0
O1—C1—C2	121.3 (4)	C18—C19—H19B	110.0
C13—C1—C2	116.8 (4)	H19A—C19—H19B	108.4
O2—C12—C11	123.6 (5)	O6—C21—C20	108.9 (5)
O2—C12—C13	116.2 (4)	O6—C21—H21A	109.9
C11—C12—C13	120.3 (5)	C20—C21—H21A	109.9
O2—C14—C15	107.2 (4)	O6—C21—H21B	109.9
O2—C14—H14A	110.3	C20—C21—H21B	109.9
C15—C14—H14A	110.3	H21A—C21—H21B	108.3
O2—C14—H14B	110.3	O15—Cl2—O14	114.5 (5)
C15—C14—H14B	110.3	O15—Cl2—O13	113.5 (6)
H14A—C14—H14B	108.5	O14—Cl2—O13	109.1 (4)
O3—C15—C14	109.9 (5)	O15—Cl2—O16	103.4 (7)
O3—C15—H15A	109.7	O14—Cl2—O16	108.8 (7)
C14—C15—H15A	109.7	O13—Cl2—O16	107.0 (7)
O3—C15—H15B	109.7	C24—C25—H25A	109.5
C14—C15—H15B	109.7	C24—C25—H25B	109.5
H15A—C15—H15B	108.2	H25A—C25—H25B	109.5
O12—Cl1—O10	108.5 (8)	C24—C25—H25C	109.5
O12—Cl1—O9	111.7 (5)	H25A—C25—H25C	109.5
O10—Cl1—O9	111.1 (5)	H25B—C25—H25C	109.5
O12—Cl1—O11	104.9 (7)	C8—C13—C12	116.8 (4)
O10—Cl1—O11	112.4 (6)	C8—C13—C1	119.5 (4)
O9—Cl1—O11	108.1 (4)	C12—C13—C1	123.7 (4)
C3—O6—C21	118.6 (5)	O8—C10—C9	123.4 (5)
C7—C2—C3	118.0 (5)	O8—C10—C11	114.8 (5)
C7—C2—C1	118.5 (5)	C9—C10—C11	121.8 (5)
C3—C2—C1	123.4 (5)	C8—C9—C10	117.4 (5)
O6—C3—C4	124.2 (5)	C8—C9—H9	121.3
O6—C3—C2	116.7 (5)	C10—C9—H9	121.3
			
O23—Tb1—O1—C1	177.8 (6)	C21—O6—C3—C4	18.6 (8)
O22—Tb1—O1—C1	−141.2 (6)	C21—O6—C3—C2	−160.6 (5)
O21—Tb1—O1—C1	0.3 (6)	C7—C2—C3—O6	175.7 (4)
O4—Tb1—O1—C1	1.7 (7)	C1—C2—C3—O6	−6.3 (7)
N1—Tb1—O1—C1	145.2 (6)	C7—C2—C3—C4	−3.5 (7)
O3—Tb1—O1—C1	72.0 (6)	C1—C2—C3—C4	174.5 (5)
O5—Tb1—O1—C1	−70.1 (6)	O2—C12—C11—C10	−177.9 (5)
O1—Tb1—O3—C16	−165.1 (4)	C13—C12—C11—C10	1.8 (7)
O23—Tb1—O3—C16	49.8 (4)	C8—O7—C7—C6	−174.9 (4)
O22—Tb1—O3—C16	104.9 (5)	C8—O7—C7—C2	6.9 (7)
O21—Tb1—O3—C16	−95.1 (4)	C3—C2—C7—O7	−179.3 (4)
O4—Tb1—O3—C16	−17.7 (4)	C1—C2—C7—O7	2.5 (7)
N1—Tb1—O3—C16	122.7 (4)	C3—C2—C7—C6	2.7 (7)
O5—Tb1—O3—C16	−35.7 (5)	C1—C2—C7—C6	−175.5 (4)
O1—Tb1—O3—C15	46.9 (4)	O7—C7—C6—C5	−178.0 (5)
O23—Tb1—O3—C15	−98.2 (4)	C2—C7—C6—C5	0.1 (7)
O22—Tb1—O3—C15	−43.1 (5)	C23—C22—O8—C10	−178.7 (5)
O21—Tb1—O3—C15	116.8 (4)	O1—Tb1—N1—C24	78.2 (16)
O4—Tb1—O3—C15	−165.7 (4)	O23—Tb1—N1—C24	−82.3 (16)
N1—Tb1—O3—C15	−25.3 (4)	O22—Tb1—N1—C24	−2.0 (16)
O5—Tb1—O3—C15	176.3 (4)	O21—Tb1—N1—C24	129.9 (15)
O1—Tb1—O4—C17	68.2 (5)	O4—Tb1—N1—C24	−124.9 (15)
O23—Tb1—O4—C17	−109.3 (5)	O3—Tb1—N1—C24	−172.1 (16)
O22—Tb1—O4—C17	−159.2 (4)	O5—Tb1—N1—C24	−22.0 (17)
O21—Tb1—O4—C17	69.6 (4)	C15—O3—C16—C17	−163.3 (5)
N1—Tb1—O4—C17	−65.2 (5)	Tb1—O3—C16—C17	45.0 (6)
O3—Tb1—O4—C17	−13.4 (4)	O6—C3—C4—C5	−177.4 (5)
O5—Tb1—O4—C17	151.6 (4)	C2—C3—C4—C5	1.7 (8)
O1—Tb1—O4—C18	−72.9 (5)	C7—C6—C5—C4	−2.1 (8)
O23—Tb1—O4—C18	109.6 (5)	C3—C4—C5—C6	1.2 (9)
O22—Tb1—O4—C18	59.6 (5)	Tb1—N1—C24—C25	74 (53)
O21—Tb1—O4—C18	−71.6 (4)	C18—O4—C17—C16	−176.2 (5)
N1—Tb1—O4—C18	153.7 (4)	Tb1—O4—C17—C16	41.0 (6)
O3—Tb1—O4—C18	−154.6 (5)	O3—C16—C17—O4	−54.6 (6)
O5—Tb1—O4—C18	10.5 (4)	C17—O4—C18—C19	178.9 (6)
O1—Tb1—O5—C19	167.7 (4)	Tb1—O4—C18—C19	−38.3 (7)
O23—Tb1—O5—C19	−45.7 (5)	C19—O5—C20—C21	−142.1 (6)
O22—Tb1—O5—C19	−121.1 (5)	Tb1—O5—C20—C21	72.4 (8)
O21—Tb1—O5—C19	98.4 (5)	C20—O5—C19—C18	163.0 (6)
O4—Tb1—O5—C19	20.6 (4)	Tb1—O5—C19—C18	−48.0 (7)
N1—Tb1—O5—C19	−101.6 (5)	O4—C18—C19—O5	55.2 (8)
O3—Tb1—O5—C19	38.6 (5)	C3—O6—C21—C20	178.7 (5)
O1—Tb1—O5—C20	−47.2 (5)	O5—C20—C21—O6	−50.1 (8)
O23—Tb1—O5—C20	99.5 (5)	O7—C8—C13—C12	176.0 (4)
O22—Tb1—O5—C20	24.1 (5)	C9—C8—C13—C12	−4.9 (7)
O21—Tb1—O5—C20	−116.5 (5)	O7—C8—C13—C1	−5.5 (7)
O4—Tb1—O5—C20	165.7 (5)	C9—C8—C13—C1	173.7 (5)
N1—Tb1—O5—C20	43.5 (6)	O2—C12—C13—C8	−178.6 (4)
O3—Tb1—O5—C20	−176.2 (4)	C11—C12—C13—C8	1.6 (7)
C7—O7—C8—C9	175.4 (4)	O2—C12—C13—C1	2.9 (7)
C7—O7—C8—C13	−5.4 (6)	C11—C12—C13—C1	−176.9 (5)
Tb1—O1—C1—C13	−90.6 (7)	O1—C1—C13—C8	−163.8 (4)
Tb1—O1—C1—C2	91.4 (6)	C2—C1—C13—C8	14.3 (6)
C14—O2—C12—C11	−14.1 (7)	O1—C1—C13—C12	14.6 (7)
C14—O2—C12—C13	166.1 (4)	C2—C1—C13—C12	−167.2 (4)
C12—O2—C14—C15	178.1 (5)	C22—O8—C10—C9	5.7 (7)
C16—O3—C15—C14	134.8 (5)	C22—O8—C10—C11	−175.9 (5)
Tb1—O3—C15—C14	−76.6 (5)	C12—C11—C10—O8	179.2 (5)
O2—C14—C15—O3	51.6 (6)	C12—C11—C10—C9	−2.4 (8)
O1—C1—C2—C7	165.3 (4)	O7—C8—C9—C10	−176.5 (4)
C13—C1—C2—C7	−12.8 (6)	C13—C8—C9—C10	4.4 (7)
O1—C1—C2—C3	−12.8 (7)	O8—C10—C9—C8	177.7 (5)
C13—C1—C2—C3	169.1 (4)	C11—C10—C9—C8	−0.6 (7)
